# Presence of Serum Antinuclear Antibodies Does Not Impact Outcomes in HBV-Related Acute-on-Chronic Liver Failure

**DOI:** 10.1155/2022/7981338

**Published:** 2022-02-18

**Authors:** Lin Lin, Bin Lin, Qing Lan, Longgen Liu, Jianchun Lu, Xiujun Zhang, Shuqin Zheng, Yuan Xue

**Affiliations:** ^1^Institute of Hepatology, The Third People's Hospital of Changzhou, Changzhou, China; ^2^Department of Pharmacy, The Third People's Hospital of Changzhou, Changzhou, China; ^3^Department of Infectious Diseases, The First People's Hospital of Jintan, Changzhou, China; ^4^Department of Infectious Diseases, The First Affiliated Hospital of Kunming Medical University, Kunming, Yunnan, China; ^5^Department of Liver Diseases, The Third People's Hospital of Changzhou, Changzhou, China

## Abstract

**Background:**

The aim of this study was to provide new insights into the prevalence of positive antinuclear antibody (ANA) in patients with HBV-related acute-on-chronic liver failure (ACLF) and its impact on clinical outcomes.

**Methods:**

A total of 116 patients with HBV-related ACLF treated at three clinical centers were retrospectively recruited. Serum concentrations of ANA were detected using the enzyme-linked immunosorbent assay kit. Multiple nuclear dots, rim-like, and centromere patterns of ANA were detected using indirect immunofluorescence assay on HEp-2 cells.

**Results:**

Among the 116 patients with HBV-related ACLF, 17 (14.66%) were ANA positive. Most patients in both ANA positive and negative groups were males (88.2% and 83.8%). Patients with negative ANA had a higher international normalized ratio, model for end-stage liver disease (MELD), and MELD-sodium scores than those with positive ANA (all *P* < 0.05). Multiple nuclear dot pattern was detected in half of the patients (8/17, 47.06%), rim-like/membranous pattern was found in six patients, and centromere pattern was detected in the last three patients. For patients with ANA (+), IgM was lower, and it was positively correlated with IgG. For patients with ANA (-), C3 was positively correlated with C4, and both C3 and C4 were negatively correlated with INR and MELD (all *P* < 0.05). In addition, TBIL, INR, WBC, and PLT, but not ANA, resulted as independent risk factors associated with 90-day mortality.

**Conclusion:**

Positive ANA is frequent in HBV-related ACLF, and it does not seem to be associated with poor outcomes, but the pathogenesis of ACLF may be different between ANA (+) and ANA (−) groups.

## 1. Introduction

Acute-on-chronic liver failure (ACLF) is a life-threatening syndrome resulting from an acute hepatic insult in patients with chronic liver diseases. Hepatitis B virus (HBV)-related ACLF (HBV-ACLF) is characterized by acute hepatic deterioration and high short-term mortality in patients with chronic hepatitis B (CHB) or liver cirrhosis (LC) [1]. Although definitions of ACLF differ among European, North America, and Asian Pacific countries, it is important to emphasize that systematic inflammation has a critical role in the pathophysiology and outcome of ACLF [2]. Moreover, according to the definition by the Asian Pacific Association for the Study of the Liver (APASL), intrahepatic precipitating events such as HBV reactivations are of the utmost importance, while extrahepatic disorders, including bacterial infections, are considered as a complication, but not a trigger of ACLF.

The issue of an antinuclear antibody (ANA) has received considerable critical attention in chronic liver disease [3, 4]. It has been reported that 21% of patients with nonalcoholic fatty liver disease (NAFLD) had positive ANA [5, 6]. Another study revealed that patients with positive ANA did not exhibit histological features of autoimmune hepatitis (AIH) or developed AIH during follow-up. Moreover, 21.8% of patients with chronic hepatitis C had positive ANA and lower sustained virological response to interferon-based therapy, while the incidence of cirrhosis, hepatocellular carcinoma, and mortality showed no significant difference from those with negative ANA [7]. It seems that long-term outcomes of NASH and chronic hepatitis C are not affected by ANA [7, 8]. To the best of our knowledge, the prevalence of ANA and its impact on clinical outcomes remain largely unknown in patients with HBV-related ACLF. Consequently, it could be useful to study whether the ANA positivity serves as an additional intrahepatic precipitating event.

This study provided new insights into the prevalence of positive ANA in patients with HBV-related ACLF and its impact on clinical outcomes.

## 2. Materials and Methods

### 2.1. Patients and the Primary Endpoint

A total of 116 patients with HBV-ACLF treated in three clinical centers were prospectively recruited between January 2015 and October 2021. ACLF was diagnosed according to the criteria from APASL [2]. Patients were excluded if they had a drug-induced liver injury, liver cancer, or coinfection with other hepatitis virus or human immunodeficiency virus. Patients with suspicious autoimmune hepatitis (AIH) or primary biliary cirrhosis (PBC) were excluded according to the simplified criteria for the diagnosis of AIH and the guideline from the British Society of Gastroenterology and UK-PBC [9–11]. Data including age, sex, survival time, alanine transaminase, aspartate transaminase, total bilirubin (TBil), international normalized ratio (INR), creatinine, HBV serologic markers, platelets count, white blood cells count, and complications, were collected.

The study was approved by the Ethics Committee of the Third People's Hospital of Changzhou according to the Declaration of Helsinki 1975, and the informed consent was obtained from all participants.

### 2.2. Enzyme-Linked Immunosorbent Assay

Serum concentrations of ANA were detected using the enzyme-linked immunosorbent assay kit (YHLO Biotech Co., Ltd., Shenzhen, Guangdong, China). Serum was initially diluted at 1 : 80 in PBS and tested; then, the positive serum was serially diluted (from 1 : 160 to 1 : 1280). Serum with a titer of >1 : 80 was reported as positive and tested for binding site. Multiple nuclear dots, rim-like, and centromere (MRC) patterns of ANA were detected using indirect immunofluorescence assay on HEp-2 cells according to the manufacturer's instructions (Medical and Biological Laboratories, Aichi-Ken, Japan). Serum concentrations of immunoglobulin (Ig) G and M and complement C3 and C4 were measured using the enzyme-linked immunosorbent assay kit (Medical system Biotechnology Co., Ltd., Ningbo, Zhejiang, China).

### 2.3. Score Systems

The model for end-stage liver disease (MELD) [12] and MELD-Na [13] scores were calculated as previously described.

### 2.4. Statistical Analysis

All data were analyzed using SPSS version 25.0 (Chicago, IL, USA). Continuous variables were expressed as median with range and compared using the Mann–Whitney *U* test. Categorical values were presented as frequencies and compared using the chi-square test. Correlation analysis was evaluated using the Spearman correlation test. Survival analysis was performed using the Kaplan–Meier curve. Independent risk factors for 90-day mortality were identified using logistic regression analysis. A two-sided *P* < 0.05 was considered statistically significant.

## 3. Results

### 3.1. Characteristics of Patients

**Figure 1:**
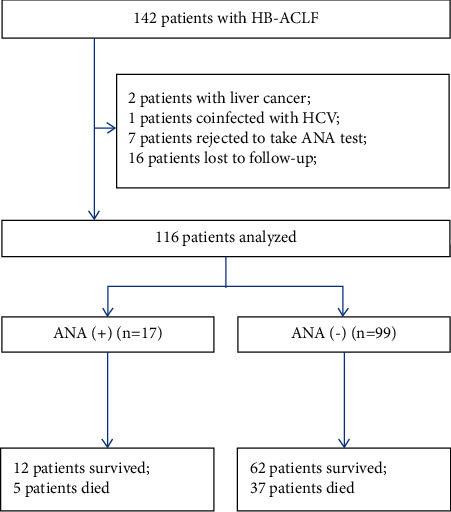
Screening of patients with acute-on-chronic liver failure and positive antinuclear antibody.

The characteristics of the 116 patients with HBV-related ACLF are given in Table 1. Among these patients, 17 (14.66%) were ANA positive (Figure 1). Most patients in both ANA positive and negative groups were males (88.2% and 83.8%, *P* > 0.99). Patients with negative ANA had higher INR, MELD, and MELD-Na scores than those with positive ANA (all *P* < 0.05). There was no significant difference in the incidence of spontaneous bacterial peritonitis (SBP) and hepatic encephalopathy (HE) between the two groups (*P* > 0.05). None of those patients from both groups were diagnosed with autoimmune hepatitis or connective tissue disease.

Among the 17 patients with positive ANA, 11 (64.71%) had a titer of 1 : 160, and the other six patients had 1 : 320. Multiple nuclear dot pattern was detected in half of the patients (8/17, 47.06%), rim-like/membranous pattern was found in six patients, and centromere pattern was detected in the last three patients.

### 3.2. Concentrations of IgG, IgM, and Complement C3 and C4

**Figure 2:**
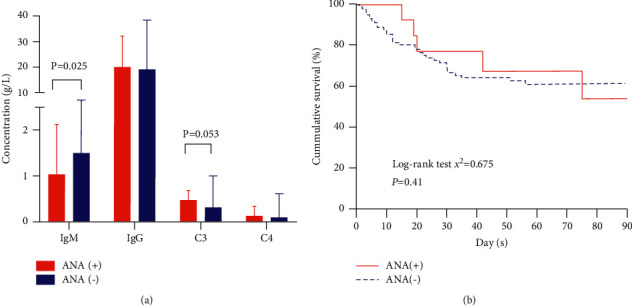
Comparison of immunoglobulin (Ig), complements, and survival between antinuclear antibody positive and negative patients with HBV-ACLF. (a) Comparison of IgG and IgM and complement C3 and C4. (b) Kaplan–Meier survival analysis between ANA (+) and ANA (−) groups during 90-day follow-up.

**Figure 3:**
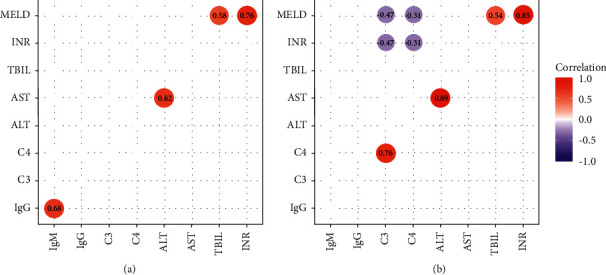
Correlation analysis between immunoglobulin (Ig), complement (C), and model for end-stage liver disease (MELD).

IgG, IgM, C3, and C4 were detected in 12 ANA (+) patients and 65 ANA (−) patients (Figure 2(a)). IgM in the ANA (+) group was significantly lower compared with that in the ANA (−) group (*P*=0.025). There was no significant difference regarding IgG, C3, and C4 between ANA (+) and ANA (−) groups (all *P* > 0.05). As shown in Figure 3, for patients with ANA (-), C3 and C4 were negatively correlated with INR (both *P* < 0.05), while the correlation was not found in patients with ANA (+). Furthermore, negative correlations between MELD score and C3 and C4 were observed in the ANA (−) group (both *P* < 0.05), but not in the ANA (+) group. Moreover, IgM was positively correlated with IgG (*γ* = 0.677, *P*=0.02) in the ANA (+) group, while C3 was positively correlated with C4 in the ANA (−) group (*γ* = 0.760, *P* < 0.01).

### 3.3. Outcomes of ANA (+) Patients with ACLF

For ANA (+) and ANA (−) patients, the 90-day mortalities were 29.4% (5/17) and 37.4% (37/99), respectively. As shown in Figure 2(b), survival analysis using the Kaplan–Meier method showed no significant differences between ANA (+) and ANA (−) groups during 90-day follow-up (*χ*^2^ = 0.675, *P*=0.41).

### 3.4. Independent Risk Factors for 90-Day Mortality

As given in Table 2, the univariate logistic analysis showed that TBIL, INR, creatinine, serum sodium, WBC, and PLT were associated with 90-day mortality. Multivariable analysis showed that TBIL, INR, WBC, and PLT (all *P* < 0.05) were independent risk factors for 90-day mortality. Next, an adjusted logistic analysis was performed using the MELD score, instead of TBIL and INR. Data showed that MELD, WBC, and PLT were independent risk factors for 90-day mortality (*P*=0.001, 0.004, and 0.023).

## 4. Discussion

The present study investigated the prevalence of positive ANA in patients with HBV-related ACLF and its impact on clinical outcomes. Our data showed that ANA was positive in 14.66% of patients with HBV-related ACLF. Patients with positive ANA had lower INR, MELD, and MELD-Na scores than those with negative ANA. For patients with ANA (+), IgM was lower, and it was positively correlated with IgG. For patients with ANA (−), C3 was positively correlated with C4, and both C3 and C4 were negatively correlated with INR and MELD. In addition, TBIL, INR, WBC, and PLT, but not ANA, resulted as independent risk factors associated with 90-day mortality.

It is speculated that positive autoantibodies in patients with chronic HBV infection may be associated with a variety of extrahepatic manifestations, while the pathogenesis remains largely unknown [14]. Over-activated CXCR5+CD4+ T cells could help B cells to secret autoantibodies and accelerate liver inflammation [14]. Biopsies in NAFLD revealed that patients with positive autoantibodies were more likely to have mild steatosis than those with negative autoantibodies [6]. In the present study, IgM was lower in patients with ANA (+), and no significant difference was found in IgG, C3, C4, and extrahepatic manifestation between ANA (+) and ANA (−) patients. INR and MELD scores were significantly lower in ANA (+) groups, indicating that ANA might be a protective factor for ACLF development. Our findings indicated that the MELD score along with WBC and PLT, but not ANA, was independently associated with the overall survival in patients with HBV-ACLF. Overall, it cannot be argued with certainty that positive ANA serves as an additional intrahepatic precipitating event in HBV-related ACLF. Moreover, considering that systemic immunosuppressive therapy is potentially harmful because it may favor the onset of infection [15], there is no evidence to support empiric steroid-based therapy for ANA (+) HBV-related ACLF.

Data from a large cohort study showed that ANA was positive in 93.4%, 49.1%, 19.1%, 13.9%, and 12.2% of patients with PBC, AIH, chronic HBV infection, chronic HCV infection, and healthy controls, respectively [16]. For patients with chronic HBV infection and positive ANA, most patients had a titer of <1 : 320, and the most frequent pattern was homogeneous [16]. A similar result was found in the present study, where ANA titers in HBV-related ACLF were low in most cases. In contrast, the main pattern in ACLF was multiple nuclear dots. The correlation between MRC patterns and liver inflammation remains unclear. It is noteworthy that multiple nuclear dot and rim-like/membranous patterns have a high specificity in patients with PBC, irrespective of the antimitochondrial antibody (AMA) status [17, 18]. AMA was routinely screened at admission, and it was negative in all these patients. Although histopathological evaluation of liver biopsy tissue could not be performed in patients with ACLF due to the coagulation disorders, there is no evidence of PBC or AIH. Of note, INR is negatively correlated with C3 and C4 in patients with ANA (−), while the correlation is not observed in the ANA (+) group; it suggests that the pathogenesis of ACLF may be different between ANA (+) and ANA (−) groups.

The present study has some limitations, such as the lack of histological characteristics and immune cells detection in patients with ANA (+) ACLF. Although IgG, the hallmark of AIH, was not high in ANA (+) patients, it could not exclude patients with co-occurrence of HBV infection and autoimmune liver diseases [19–21]. Considering that the sample size was not large, future large-scale prospective studies are needed to confirm these findings.

## 5. Conclusions

Positive ANA is frequent in HBV-related ACLF, and it does not seem to be associated with poor outcomes, but the pathogenesis of ACLF may be different between ANA (+) and ANA (−) groups.

## Figures and Tables

**Table 1:** Characteristics of patients with ACLF.

Variables	ANA positive (*n* = 17)	ANA negative (*n* = 99)	*Z* or *χ*^*2*^	*P* value
Age (years)	50.0 (33.0–59.0)	51.0 (41.0–59.0)	−0.859	0.39
Male, *n* (%)	15 (88.2)	83 (83.8)	0.214	>0.99
HBeAg, *n* (%)	6 (35.3)	32 (32.3)	0.058	0.81
ALT (U/L)	322.0 (156.3–991.3)	272.3 (87.0–690.0)	−0.972	0.33
AST (U/L)	292.8 (120.3–418.7)	178.0 (91.4–419.0)	−1.296	0.20
TBil (*μ*mol/L)	288.8 (201.8–395.8)	302.4 (212.5–377.7)	−0.121	0.90
Creatinine (*μ*mol/L)	67.1 (55.8–80.5)	64.2 (54.4–83.9)	−0.293	0.77
Platelet (*E* + 09/L)	102.0 (59.5–181.0)	98.0 (70.0–134.0)	−0.894	0.37
WBC (*E* + 09/L)	7.1 (5.6–9.6)	6.0 (4.8–9.3)	−1.300	0.19
Neutrophils (*E*+09/L)	4.8 (3.6–6.5)	4.0 (2.9–6.0)	−1.261	0.21
Serum sodium (mmol/L)	139.0 (136.7–140.2)	137.6 (134.6–139.8)	−1.405	0.16
INR	1.8 (1.6–2.1)	2.1 (1.8–2.7)	−2.799	<0.01
MELD score	24.0 (21.5–26.0)	26.0 (24.0–29.0)	−2.449	0.01
MELD-Na score	25.0 (21.5–26.0)	27.0 (24.0–31.0)	−2.429	0.01
Cirrhosis, *n* (%)	10 (58.8%)	58 (58.1%)	0.001	0.99
SBP, *n* (%)	5 (29.4%)	29 (29.3%)	0.001	0.99
HE, *n* (%)	7 (41.2%)	44 (44.4%)	0.063	0.80
Death (%)	5 (29.4%)	37 (37.4%)	0.398	0.53

Data were expressed as median (IQR) for continuous variables and *n* (%) for categorical values and were compared using the Mann–Whitney *U* test or chi-square test. ACLF, acute-on-chronic liver failure; ANA, antinuclear antibodies; ALT, alanine aminotransferase; AST, aspartate aminotransferase; TBil, total bilirubin; ALP, alkaline phosphatase; GGT, gamma glutamyl transferase; CHE, cholinesterase; INR, international normalized ratio; HBeAg, hepatitis B e antigen; WBC, white blood cell; MELD, model for end-stage liver disease; SBP, spontaneous bacterial peritonitis; HE, hepatic encephalopathy.

**Table 2:** Risk factors for 90-day mortality in patients with ACLF.

Baseline variables	Univariate			Multivariate		
	Odds ratio	95% CI	*P*	Odds ratio	95% CI	*P*
Age (years)	0.988	0.949–1.029	0.57			
Male	1.211	0.242–6.053	0.82			
LC	1.312	0.338–5.094	0.70			
HE	0.537	0.189–1.522	0.24			
SBP	0.592	0.150–2.339	0.45			
ANA (+)	0.938	0.207–4.259	0.93			
ALT	1.000	0.997–1.002	0.66			
AST	1.001	0.999–1.004	0.36			
TBil	1.004	0.999–1.009	0.09	1.005	1.000–1.009	0.04
INR	2.126	0.889–5.084	0.09	2.559	1.215–5.387	0.01
Creatinine	1.017	0.999–1.035	0.06			
Serum sodium	0.891	0.780–1.016	0.09			
PLT	0.982	0.966–1.000	0.04	0.983	0.971–0.996	<0.01
WBC	1.216	0.978–1.510	0.08	1.307	1.103–1.550	<0.01

ACLF, acute-on-chronic liver failure; 95% CI, 95% confidence interval; LC, liver cirrhosis; ALT, alanine aminotransferase; AST, aspartate aminotransferase; ANA, antinuclear antibodies; CHE, cholinesterase; TBil, total bilirubin; INR, international normalized ratio; PLT, platelet; WBC, white blood cell.

## Data Availability

The data used to support the findings of this study are available from the corresponding author upon request.

## Abbreviations

HBV: Hepatitis B virus
ACLF: Acute-on-chronic liver failure
LC: Liver cirrhosis
CHB: Chronic hepatitis B
ANA: Antinuclear antibody
APASL: Asian Pacific Association for the Study of the Liver
NAFLD: Nonalcoholic fatty liver disease
AIH: Autoimmune hepatitis
MRC: Multiple nuclear dots, rim-like, and centromere
HBeAg: Hepatitis B e antigen
MELD: Model for end-stage liver disease
ALT: Alanine aminotransferase
AST: Aspartate aminotransferase
TBil: Total bilirubin
INR: International normalized ratio
WBC: White blood cells
SBP: Spontaneous bacterial peritonitis
HE: Hepatic encephalopathy.
